# The Regulatory Role of MicroRNAs in EMT and Cancer

**DOI:** 10.1155/2015/865816

**Published:** 2015-03-25

**Authors:** Apostolos Zaravinos

**Affiliations:** Department of Laboratory Medicine, Karolinska Institutet Huddinge, 171 77 Stockholm, Sweden

## Abstract

The epithelial to mesenchymal transition (EMT) is a powerful process in tumor invasion, metastasis, and tumorigenesis and describes the molecular reprogramming and phenotypic changes that are characterized by a transition from polarized immotile epithelial cells to motile mesenchymal cells. It is now well known that miRNAs are important regulators of malignant transformation and metastasis. The aberrant expression of the miR-200 family in cancer and its involvement in the initiation and progression of malignant transformation has been well demonstrated. The metastasis suppressive role of the miR-200 members is strongly associated with a pathologic EMT. This review describes the most recent advances regarding the influence of miRNAs in EMT and the control they exert in major signaling pathways in various cancers. The ability of the autocrine TGF-*β*/ZEB/miR-200 signaling regulatory network to control cell plasticity between the epithelial and mesenchymal state is further discussed. Various miRNAs are reported to directly target EMT transcription factors and components of the cell architecture, as well as miRNAs that are able to reverse the EMT process by targeting the Notch and Wnt signaling pathways. The link between cancer stem cells and EMT is also reported and the most recent developments regarding clinical trials that are currently using anti-miRNA constructs are further discussed.

## 1. Epithelial to Mesenchymal Transition (EMT)

The epithelial to mesenchymal transition is a unique process that describes the molecular reprogramming and phenotypic changes characterized by a transition from polarized immotile epithelial cells to motile mesenchymal cells, thus leading to increased motility and invasion. This transition is characterized by a decrease in the expression of proteins that enhance cell-cell contact such as E-cadherin and *γ*-catenin, as well as an increase in the expression of mesenchymal markers such as vimentin, N-cadherin, and fibronectin, as well as the activity of some matrix metalloproteinases. EMT was initially identified during embryogenesis and was later shown to be involved in neural crest [1] and heart-valve formation [2] and palate fusion [3]. More recently, EMT was shown to play a critical role in tumor invasion and metastasis [4]. EMT is categorized into developmental (Type I), fibrosis and wound healing (Type II), and cancer (Type III) [5]. Mesenchymal to epithelial transition (MET) [6] is the reverse process and also plays an important role in the formation of the kidney nephron epithelium. It is now widely known that EMT constitutes an early metastatic step [7], where cells that have undergone EMT can detach from the primary tumor, invade through the basement membrane into the circulation, and converse back to an epithelial phenotype to form a metastasis at a distant secondary site [8].

## 2. Signaling Pathways Involved in EMT

**Figure 1:**
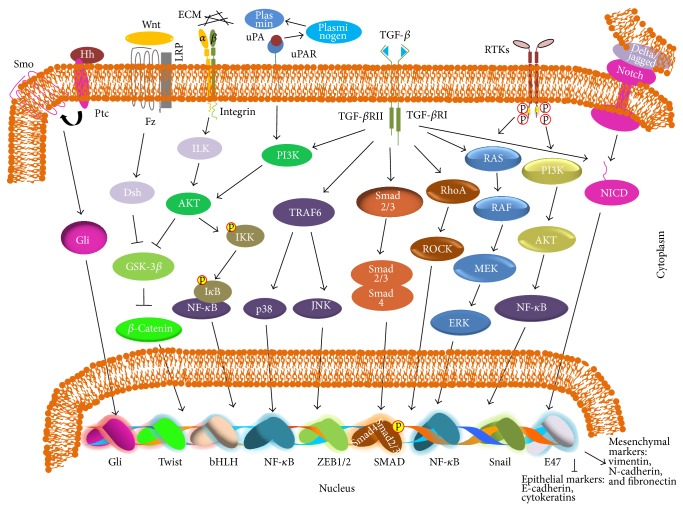
Major interconnected signaling pathways that regulate EMT. The Smad pathway for TGF-*β* signaling acts through the formation of a complex between Smad 2/3 and Smad 4. The complex then moves to the nucleus and stimulates the transcription of target genes. Sharp arrows denote activation/upregulation and blunt arrows denote inhibition/downregulation. Fz: frizzled receptors; Gli: glioma-associated oncogene family of transcription factors; GSK-3b: glycogen synthase kinase; Hh: hedgehog; PI3K: phosphatidylinositol-3-kinase; ILK: integrin-linked kinase; LRP: low-density lipoprotein receptor-related protein; p38 MAPK: mitogen-activated protein kinase; Ptc: patched receptor for Hh signaling; SMO: smoothened; TGF-*β*: transforming growth factor *β*; uPAR: urokinase plasminogen activator receptor.

Various signaling pathways can induce EMT and include key molecules such as transforming growth factor beta (TGF-*β*), growth factors that act through tyrosine kinase receptors (RTKs), like platelet-derived growth factor (PDGF) and fibroblast growth factor receptors (FGFRs) [4, 9], and the proteins nuclear factor kappa-light-chain-enhancer of activated B cells (NF-*κ*B), Wnt (*wingless integrated*), and Notch and hedgehog (Hh) proteins [10] (Figure 1). These signaling pathways stimulate transcription factors like Snail, basic helix-loop-helix (bHLH), zinc finger E-box-binding homeobox1/2 (ZEB1/2), and NF-*κ*B, among others, that repress epithelial gene expression and act as activators of EMT [11]. These proteins bind to the promoter of E-cadherin silencing its expression. E-cadherin is a central component of the adherens junction complex, responsible for the calcium-dependent cell-cell adhesion and the maintenance of the cytoskeletal organization. Its loss is a causal factor in cancer progression. Transcriptional repression of E-cadherin is an important emerging mechanism through which the gene is downregulated during tumor progression and several transcription factors, among them Snail, Slug/Snail2, ZEB1, ZEB2, and E47, directly bind to its promoter and repress its transcription. EMT is induced through various channels. Many of these E-cadherin repressors are induced by the stimulation of the TGF-*β* pathway and they can further repress the transcription of other cell polarity and adhesion genes [12] (Figure 1).

TGF-*β* is a major inducer of EMT [13–15]. It binds to its receptors (TGF-*β*RI, TGF-*β*RII, and TGF-*β*RIII) leading to the activation through phosphorylation of Smad 2 and Smad 3. These in turn form trimers with Smad 4 and the complex is translocated into the nucleus where it regulates the expression of TGF-*β* target genes along with other DNA binding factors, like Snail, ZEB, and Twist [16, 17]. The result is the downregulation of epithelial markers (E-cadherin and cytokeratins) and the upregulation of mesenchymal markers (vimentin, N-cadherin, and fibronectin). The activation of RTKs and their downstream signaling effectors such as MAPK or PI3K is crucial for an increased rate of cell proliferation in epithelial cells. Signaling via either MAPK or PI3K along with TGF-*β* is also necessary and sufficient to regulate EMT [18]. Crosstalk of TGF-*β* with other signaling pathways like Notch, Wnt/*β*-catenin, NF-*κ*B, and RTKs can also induce EMT which further helps in maintaining the mesenchymal phenotype of metastatic tumor cells [4, 9, 10, 19] (Figure 1).

Wnt signaling is also important for the regulation of EMT and diverse cell functions via canonical (*β*-catenin) or noncanonical pathways [20]. The formation of the Wnt-Fz-LRP complex through the binding of wnt1 and wnt3 ligands to their receptors, Frizzled (Fz) and LDL-receptor-related protein 5/6 (LRP 5/6), initiates the canonical pathway. Without the Wnt signaling pathway, cytoplasmic *β*-catenin forms a complex with Axin, adenomatous polyposis coli (APC), glycogen synthase kinase-3b (GSK-3*β*), and casein kinase 1 (Ck1) [21]. When the cell receives Wnt signals, LRP5/6 and Fz form a complex. These structures affect the stabilization of *β*-catenin, its translocation to the nucleus, and its protein accumulation. In the nucleus, *β*-catenin forms a complex with T-cell factor/lymphoid enhancer factor (TCF/LEF) initiating the transcription of Wnt target genes, including Snail1 [21, 22]. During EMT, Smad 2 and Smad 4 influence Wnt signaling to repress E-cadherin expression in medial-edge epithelial cells (Figure 1).

Another important pathway in EMT is Hedgehog (Hh) signaling. Hh is a major regulator of cell proliferation, differentiation, and tissue polarity. The Hh family consists of three Hh proteins, including Sonic Hedgehog, Desert Hedgehog, and Indian Hedgehog [23]. Binding of Hh ligands to their receptors causes activation of a family of transcriptional factors through complex cascades. This leads to the upregulation of Wnt protein *β*-catenin and bone morphogenic protein accumulation in the cytoplasm. Wnt and Hh signaling are both mediated by the G-protein coupled Frizzled receptors, and both pathways prevent phosphorylation-dependent proteolysis of *β*-catenin. In addition, the molecules involved in Wnt signaling such as GSK-3*β* also regulate Hh signaling, suggesting crosstalk between the two potential pathways (Figure 1).

The Notch signaling pathway is also considered an important regulator for EMT induction, despite several reports that Notch signaling is insufficient to completely induce EMT and it requires crosstalk with other signaling molecules [20]. The Notch pathway is initiated through interactions between the Notch receptor and ligands on adjacent cells. Four Notch receptors (1–4) and five ligands (Dll-1, Dll-3, Dll-4, Jagged-1, and Jagged-2) have been shown to exist in mammals [24, 25]. Notch signaling is initiated through ligand binding to an adjacent receptor. Subsequently, the intramembrane Notch receptor (NICD) is cleaved by *γ*-secretase. The released NICD then translocates to the nucleus and interacts with C-protein binding factor 1/Suppressor of Hairless/Lag-1 (CBF1/Su (H)/Lag 1) [25, 26] and acts as an activator of target genes, including Hes and Hey (Figure 1). The Notch pathway maintains a balance between cell proliferation, differentiation, and apoptosis and plays an important role in determining cell fate and maintaining progenitor cell population. Notch signaling requires coordination with other signals to promote EMT. TGF-*β* increases Notch activity through Smad 3, subsequently promoting Slug expression which suppresses E-cadherin [27]. Slug-induced EMT is accompanied by the activation of *β*-catenin and resistance to anoikis. Wnt and Notch pathways have also been shown to cross-link between each other in order to induce a tumorigenic phenotype [19, 28].

E-cadherin is anchored to the cytoskeleton via *β*-catenin, a cytoplasmic plaque protein [29]. In loss of cell adhesion, during invasion, E-cadherin is endocytosed and *β*-catenin is released. In normal and noninvasive cells, *β*-catenin is usually localized to the cell membrane. In cells undergoing EMT, *β*-catenin is located in the cytoplasm. This cytosolic *β*-catenin translocates to the nucleus to promote transcription of genes that induce EMT (Figure 1).

The integrin-linked kinase (ILK) pathway has also been reported to induce EMT. Integrins are heterodimeric adhesion receptors composed of *α* and *β* subunits. There are 18 *α* and 8 *β* subunits that variously combine into 24 different integrins. Integrins bind to ligands, including collagens, laminins, and fibronectin in the ECM. Ligand-bound integrins induce several signaling cascades that control cell polarity, motility, survival, shape, proliferation, and differentiation [30] (Figure 1).

uPAR (urokinase-type plasminogen activator receptor) signaling also plays a role in EMT [31]. Urokinase was originally isolated from human urine but can also be present in several other locations including the ECM. The main physiological substrate for urokinase plasminogen activator (uPA) is plasminogen. When uPA, a serine protease, binds to uPAR, plasminogen is activated to form plasmin (Figure 1). Activation of plasmin triggers a proteolytic cascade that can participate in ECM remodeling, degrading components of the basement membrane, and hence allowing cells to move across and through these barriers [31, 32]. Binding of uPA to uPAR can induce EMT through activating a number of cell-signaling factors, including PI3K, Src family kinases, Akt, ERK/MAPK, and myosin light chain kinase [33, 34]. Among them, only the PI3K/AKT pathway has been studied in uPAR signaling in EMT. Activation of PI3K signaling catalyzes the formation of phosphatidylinositol 3,4,5-phosphate, which can influence cell morphology through its effect on actin cytoskeleton reorganization and migration [32]. Another mechanism by which PI3K may also be involved is through the activation of AKT, which can promote cell invasion [32] and regulate the activity of transcription factors like NF-*κ*B that binds to the DNA sequence and induce EMT [35].

## 3. MicroRNAs (miRNAs)

miRNAs are small (19–25 nucleotides long) noncoding, single-stranded RNAs that control gene expression by targeting mRNA transcripts and leading to their translational repression or degradation, according to the level of complementarity with them [36, 37]. To date, over 2,500 potential human miRNAs have been recorded in miRBase v20 [36] and their number is increasing rapidly. Taking into account that one miRNA can target many mRNA transcripts and that one mRNA transcript can be targeted by many miRNAs, it can be roughly estimated that ~10–40% of the mRNA sequences are targeted by miRNAs in human [38]. Therefore, there is a great need to validate the targets of newly discovered miRNAs.

miRNAs can be both differentially and temporally expressed in a tissue- and developmental-specific mode [39–42]. Various miRNA signatures can accurately distinguish tumor from normal tissue, as well as various cancerous subtypes among them [39]. Furthermore, it is now well established that miRNAs can serve as candidate biomarkers for diagnostic and prognostic purposes [40, 43]. miRNA genes are usually intronic and clustered and are transcribed by RNA polymerase II producing a primary miRNA (pri-miRNA) of several kb in length. Pri-miRNAs are cleaved at specific sites in the nucleus (resulting in pre-miRNAs) and in the cytoplasm (resulting in mature miRNAs), by the RNases Drosha by Dicer, respectively [44]. Mature miRNAs are then activated by binding to the Argonaute 2 (AGO2) in the miRNA-induced silencing complex (miRISC) [45]. In particular, the “seed” sequence across nucleotides 2–8 at the 5′ end of the mature miRNA binds its complementary sequence within the 3′ UTRs of its target mRNA transcripts. Perfect complementarity between the miRNA and its mRNA target often leads to mRNA deadenylation and degradation, whereas imperfect complementarity leads to the inhibition of translation [46]. Mature miRNAs can also regulate gene expression by binding to the 5′ UTR of their target genes or their coding regions (CDS) [47]. CDS-based sites are more effective at inhibiting translation, whereas sites in the 3′ UTR are more specialized for promoting degradation [47]. Many miRNAs are now known to suppress various important cancer-related genes, therefore, acting as oncogenes or tumor suppressors [48]. Several miRNAs have been identified to regulate EMT.

## 4. The miR-200 Family and Its Metastasis Suppressive Role in Cancer

The miR-200 family is composed of 5 miRNA sequences: miR-200a, miR-200b, miR-200c, miR-141, and miR-429, clustered and expressed as two separate polycistronic pri-miRNA transcripts: miR-200a, miR-200b, and miR-429 (chromosome 1) and miR-200c and miR-141 (chromosome 12) [49]. The miR-200 family plays an essential role in EMT suppression mainly through targeting ZEB and its function was recently reviewed [50–52]. The role of miR-200 in EMT and tumor progression has been linked to several cancers [53–63]. Gregory et al. found markedly low miR-200 levels in cells that had undergone EMT, in response to TGF-*β*. The enforced miR-200 expression alone was also shown to be sufficient to prevent TGF-*β*-induced EMT and miR-200 inhibition was sufficient to induce EMT. Conversely, ectopic expression of the miR-200 members in mesenchymal cells initiated MET [64]. Moreover, in invasive breast cancer, the lack of miR-200 expression was positively correlated with absent E-cadherin [64]. Further supporting these results, both miR-200 clusters were shown to be clearly downregulated in a TGF-*β* inducible mouse model of mammary tumor with EMT. The overexpression of miR-200 members caused E-cadherin upregulation and inhibited EMT via targeting the transcription factors ZEB1 and ZEB2 [65].

The metastasis suppressive role of the miR-200 family was further studied in tumor cell lines derived from mice that develop metastatic lung adenocarcinoma owing to expression of mutant K-ras and p53. Following a TGF-*β* treatment, the cells entered EMT and this transition was entirely miR-200 dependent [63]. Furthermore, in non-small-cell lung cancer (NSCLC) cell lines, miR-200 was correlated with EMT markers, distinguishing between those lines that derived from primary lung tumors and the ones that originated from metastatic lesions [63]. In metastatic NSCLC cells, the reexpression of miR-200 downregulated genes that are involved in metastasis signaling and proliferation, such as DLC1, ATRX, HFE, HNRNPR3, HFE, and ATRX [66]. The miR-200 expression was also demonstrated to change the tumor microenvironment and inhibit EMT and metastasis, in lung adenocarcinoma [67]. miR-200 was further reported to enhance macroscopic metastases in mouse breast cancer cell lines [56]. Dykxhoorn et al. [56] reported that, for some tumors, tumor colonization at metastatic sites might be enhanced by MET, which suggests that the epithelial nature of a tumor does not predict metastatic outcome.

## 5. The TGF-***β***/ZEB/miR-200 Regulatory Network

**Figure 2:**
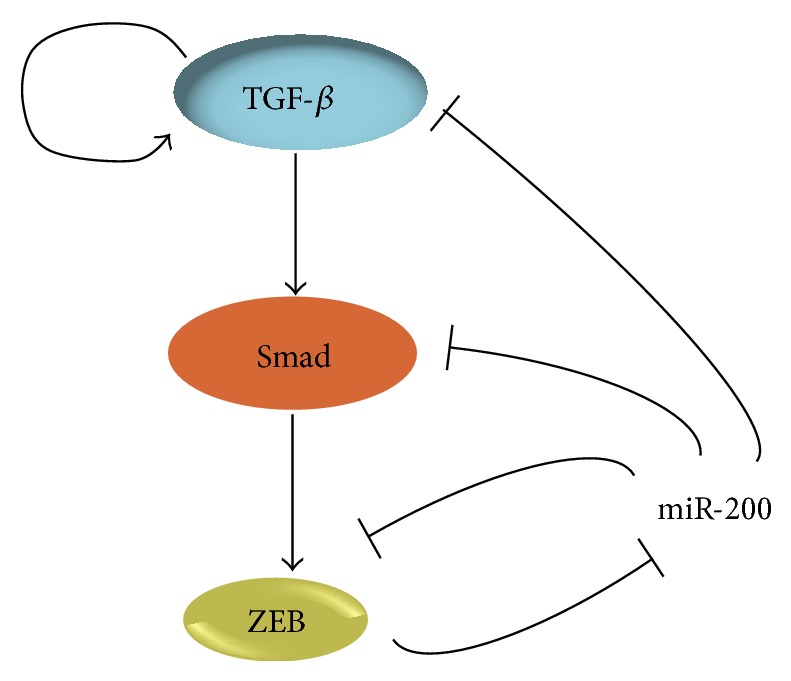
The TGF-*β*/ZEB/miR-200 regulatory network.

Gregory et al. recently demonstrated the existence of an autocrine TGF-*β*/ZEB/miR-200 signaling regulatory network that controls the plasticity between the epithelial and mesenchymal states of the cells. Strong correlation was reported between the ZEB1/2 and TGF-*β* and negative correlations were detected between miR-200 and TGF-*β*, as well as between miR-200 and ZEB1/2, in invasive ductal carcinomas [68]. ZEB1/2 can induce EMT by repressing various epithelial genes [69]. The TGF-*β* signaling pathway is a central activator of ZEB1/2, indicating that they are important intracellular mediators of the TGF-*β*-induced EMT. The crosstalk between the ZEB/miR-200 axis and several signal transduction pathways activated at different stages of tumor development was also reviewed recently [70]. ZEB1/2 and the miR-200 family are involved in a double-negative feedback loop, which controls EMT and MET programs both in development and tumorigenesis [70]. On one hand, the miR-200 members target and suppress ZEB1/2 and promote epithelial differentiation [64, 71, 72]. On the other hand, ZEB1 knockdown can enhance miR-200 [73] (Figure 2). This was supported when it was found that the common promoter region of the miR-200 members includes highly conserved ZEB-binding sites, through which ZEB factors control the transcription of the miR-200 family [73, 74]. ZEB downregulation leads to the enhancement of an epithelial pattern of gene expression through induction of the miR-200 members. On the contrary, ZEB expression induces a mesenchymal pattern of gene expression through miR-200 suppression. This feedback loop was shown to play important roles in the stabilization of cellular differentiation in response to prevalent extracellular cues [75]. In gastric cancer, miR-200b can control metastasis by regulating the expression of ZEB2 [76]. Cong et al. found inversely related expression levels between miR-200a and ZEB1/2 in gastric adenocarcinoma tissue arrays. The upregulated miR-200a expression was also found to increase E-cadherin and suppress the Wnt/*β*-catenin pathway by targeting ZEB1/2 in gastric adenocarcinoma, thus delaying tumor growth in vivo [77].

The permanence of the mesenchymal phenotype following EMT is sustained by TGF-*β*-containing autocrine loops (Figure 2) [78–80]. TGF-*β* can induce its own autocrine production, cooperating with the RAF-MAPK and the *β*-catenin signaling pathways that trigger EMT [81, 82]. TGF-*β*2 is a predominant target of the miR-200 family and the relief of miR-200-mediated inhibition of TGF-*β*2 increases the autocrine effect of TGF-*β* [73] (Figure 2). Therefore, the interconnection among TGF-*β*, miR-200, and ZEB can explain the reversibility of the mesenchymal phenotype. Nevertheless, the mechanism by which the ZEB/miR-200 loop activates autocrine TGF-*β* signaling is not clear enough. One explanation is that Smads bind to the promoter of ZEB and induce its TGF-*β*-mediated transcription [68, 83] (Figure 2). On the other hand, the autocrine TGF-*β* signaling was shown to induce a reversible methylation of the miR-200 loci, through the recruitment of histone-modifying complexes by ZEB proteins. Smad and TGF-*β* were further shown to be direct targets of miR-200 [84] (Figure 2). Recently, microenvironment-dependent cues were suggested to trigger miRNA-regulated feedback loops facilitating the switch between EMT and MET [85]. Although many TGF-*β*-induced pathways are necessary for the induction and maintenance of EMT [86], it is not clear how they control the expression of ZEB.

## 6. Other EMT-Regulating miRNAs

### 6.1. miRNAs That Control EMT Transcription Factors

Many other miRNAs can directly target EMT transcription factors. miR-205 acts synergistically with miR-200 members in order to suppress ZEB and lead to MET [64]. In mammary gland cells, miR-205 maintains the epithelial differentiation [87–89]. In prostate cancer, miR-29b suppresses metastasis by regulating EMT signaling [90]. Also, miR-30a is downregulated during EMT in murine hepatocytes [91]. Furthermore, in NSCLC, Snai1 is posttranscriptionally targeted by miRNA-30a [92]. In hepatoma cells, miR-148a can negatively regulate Met/Snail signaling and prevent EMT and metastasis [93]. Snail and miR-34 form another double feedback loop, in which the first binds to E-boxes that are located within the promoter of the miR-34 gene, thereby leading to the transcriptional repression of the second [94]. In TGF-*β*-induced EMT, increased Snail expression can suppress miR-34. A novel miR-203/SNAI1 feedback loop was also reported in breast cancer [95]. These double-feedback loops can enhance the activation of EMT and control the balance between the two states of the cell (epithelial and mesenchymal). Recently, a novel EMT network integrating the negative feedback loops, miR-203/SNAI1 and miR-200/ZEB, was proposed to function as a switch that controls the plasticity of epithelial cells during their differentiation and the progression of cancer [95]. In metastatic breast cancer cells, the expression of miR-10b was shown to be induced by the transcription factor Twist, which binds directly to the putative promoter of miR-10b [96]. The Twist-induced miR-10b thereby inhibits translation of the mRNA encoding homeobox D10, resulting in the increased expression of RHOC, a well-characterized prometastatic gene [96].

### 6.2. miRNAs Targeting Components of the Cell Architecture

Many miRNAs interfere with EMT by targeting components of the cell architecture [97–102]. A direct transcriptional target of the TGF-*β*/Smad 4 signaling is miR-155 [103]. Its knockdown can suppress TGF-*β*-induced EMT and the dissolution of tight junctions, as well as cell migration and invasion [99]. Furthermore, the ectopic expression of miR-155 can reduce the expression of RhoA (Ras homolog gene family, member A) protein, a small GTPase protein known to regulate the actin cytoskeleton in the formation of stress fibers and disrupt the formation of tight junctions [99].

In colon cancer cells upon treatment with TGF-*β*, miR-21 and miR-31 were induced and could lead to enhanced cellular motility and invasiveness. Their elevated expression was associated with lymph node positivity and the development of distant metastases in patients suffering from colorectal cancer [104]. In the progression of colorectal cancer, both miRNAs could promote TGF-*β*-induced EMT, by repressing the translation of TIAM1 (T-cell lymphoma invasion and metastasis 1), a guanidine exchange factor of the Rac GTPase [101]. In established metastases, the activation of miR-31 was shown to lead to regression of metastasis and prolongation of patient survival. Furthermore, its induction could reduce the metastatic potential of cancer cells, via targeting RhoA [105].

In human breast cancer cells, miR-9 is upregulated and directly represses cadherin-1 (CDH1), a calcium-dependent protein involved in mechanisms regulating cell-cell adhesions, mobility, and proliferation of epithelial cells. CDH1 repression leads to increased cell motility and invasiveness [106]. Furthermore, the loss of E-cadherin liberates *β*-catenin which translocates into the nucleus and activates prometastatic genes, such as VEGF [107], which in turn leads to increased tumor angiogenesis. During EMT, E-cadherin suppression is often accompanied by upregulation of N-cadherin. On the other hand, miR-194 negatively regulated the expression of N-cadherin. However, miR-194 expression is attenuated in advanced stage gastric cancer cells, whereas in mesenchymal hepatic cancer cells, miR-194 expression is enhanced and N-cadherin, cell migration, invasion, and metastasis are reduced [108].

In hepatocellular carcinoma cells (HCC), miR-490-3p enhances cell proliferation, migration, and invasion abilities and stimulates EMT, via targeting ERGIC3 (ER-Golgi intermediate compartment protein 3), also known as endoplasmic reticulum-localized protein/ERp43. ERGIC3 is a protein with a possible role in transport between ER and Golgi [109]. Overexpression of ERp43 was shown to accelerate cell growth and to inhibit ER stress-induced cell death, while its downregulation decreased the rate of cellular proliferation and enhanced cell death [110]. ERGIC3 was also shown to stimulate cell migration and their ability to invade [109]. The same authors found that, in HCC cells, miR-490-3p led to increased cell proliferation, migration, and invasion, thus contributing to EMT. ERGIC3 was shown to be directly targeted by miR-490-3p, which unexpectedly increased its mRNA and protein levels [109].

Furthermore, miR-29a was found to be the most highly upregulated miRNA during EMT in response to TGF-*β* in the murine mesenchymal, metastatic RasXT cells relative to epithelial EpRas cells. miR-29a can target tristetraproline (TTP), a protein involved in the degradation of messenger RNAs with AU-rich 3′-untranslated regions, and led to EMT and metastasis in cooperation with oncogenic Ras signaling [100]. All these results demonstrate the ability of miRNAs to regulate EMT in cancer progression, via the targeting of components of the cell architecture.

### 6.3. miRNAs Targeting Multiple EMT/MET Components

Some miRNAs regulate EMT by targeting either the receptors that accept signals from EMT inducers or multiple EMT/MET components. TGF-*β*RII and Snail2 were demonstrated to be directly targeted by miR-204. A reduction in miR-204 expression led to reduced levels of claudins 10, 16, and 19 [111]. miR-204 has dual roles in maintaining the integrity of the epithelium, since it can also target Snail, which is rapidly induced by TGF-*β* signaling during EMT [111].

The Eph tyrosine kinase receptor A4 (EphA4) regulates MET of the paraxial mesoderm during somite morphogenesis [112]. It also promotes cell proliferation and migration through an EphA4-FGFR1 signaling pathway [113]. In HCC, miR-10a targets EphA4 and regulates the metastatic properties of the cancer cells [114]. EphA4 knockdown phenocopied the effect of miR-10a and its ectopic expression restored the effect of miR-10a on migration, invasion, and adhesion in HCC cells [114].

The induction of EMT in luminal breast cancer cells involves the downregulation of miR-200 members and the upregulation of the miR-221 family [115]. Luminal cells expressing miR-221/222 gained a more mesenchymal phenotype and increased cell motility and invasiveness [115], whereas the inhibition of miR-221/222 in basal-like cells promoted MET [116]. Providing a functional link between miR-221/222 expression and E-cadherin repression in breast cancer cells, miR-221/222 could directly target trichorhinophalangeal 1 (TRPS1), a transcriptional repressor of ZEB2 [115, 117]. miR-221/222 can also repress Dicer, a key protein in the maturation of miRNAs [118].

In squamous cells, miR-138 was shown to regulate EMT by targeting components of the EMT pathways, such as RhoC (Ras homolog gene family, member C) and ROCK2 (Rho-associated, coiled-coil containing protein kinase 2) [119]. Its enhanced expression could repress RhoC and ROCK2 in tongue squamous cell carcinoma, leading to diminished cellular migration and invasion [120]. Additionally, miR-138 regulates EMT either via direct targeting vimentin or via targeting ZEB2, which in turn regulates the transcription activity Cadherin-1. Furthermore, it can regulate EMT through targeting the epigenetic regulator enhancer of zeste homologue 2 (EZH2), which in turn modulates its gene silencing effects on downstream genes (e.g., E-cadherin) [121]. miR-101 was recently reported to act as a crucial tumor suppressor which suppresses cell proliferation, invasiveness, and self-renewal in aggressive endometrial cancer cells via the modulation multiple critical oncogenes [122]. The axis miR-101-EZH2/MCL-1/FOS was proposed to be a potential therapeutic target for endometrial cancer [122].

### 6.4. miR-200 Members Regulate the Notch and Wnt Signaling Pathways

Recently, a coupling between the ZEB/miR-200 axis and the Notch pathway was established in cancer [123–125]. ZEB1 was shown to trigger Notch signaling by stabilizing the expression of Jagged1, Maml2, and Maml3, through inhibition of the miR-200 members [124]. This suggests that the ZEB-dependent downregulation of miR-200 feeds back positively on ZEB expression and results in the stabilization of a mesenchymal cell phenotype [124].

**Figure 3:**
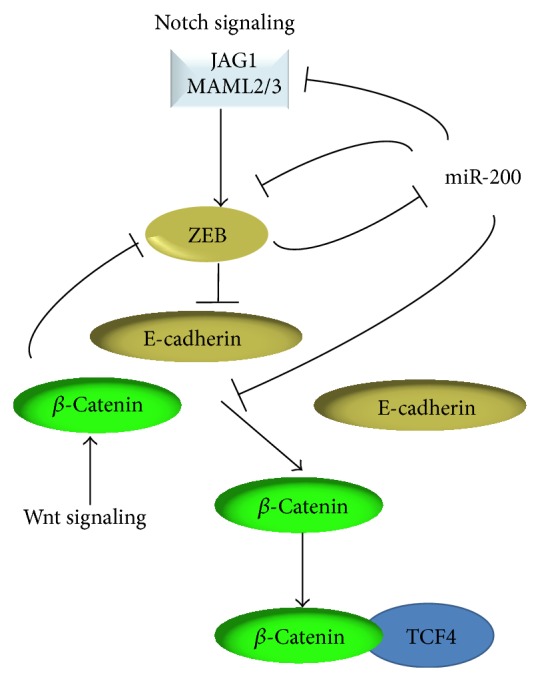
Regulation of the Notch and Wnt signaling pathways by miR-200.

Furthermore, a functional link was recently established between the canonical Wnt pathway and ZEB1, demonstrating that ZEB1 is a direct transcriptional target of *β*-catenin in colon cancer cells [126, 127]. However, the ZEB2/Wnt relationship in colon cancer yet remains unclear. Wnt signaling is also connected with the ZEB/miR-200 network in cancer. miR-200a was reported to downregulate *β*-catenin-mediated transcription via targeting either ZEB or *β*-catenin, thus downregulating the activation of Wnt/*β*-catenin signaling [128] (Figure 3). In contrast, miR-200b and miR-200c have no effect on *β*-catenin.

### 6.5. The ZEB/miR-200 Network and p53 Family Members

**Figure 4:**
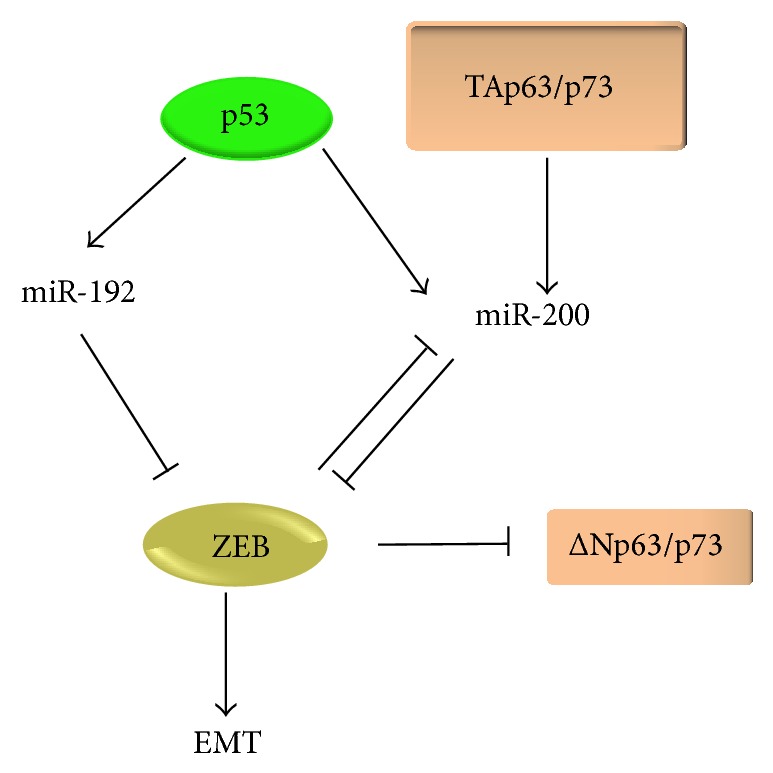
The ZEB/miR-200 network and the p53 family members.

The p53 transcription factor can induce or repress a large set of genes and miRNAs [129, 130]. The p53 and its homologs p63 and p73 are well involved in tumor metastasis and tumor progression [131–133]. The p63 and p73 members exist as full-size proteins (TAp63 and TAp73) or truncated forms (ΔNp63 and ΔNp73) that lack the transcriptional activation domain. In early studies, it was demonstrated that ΔNp63/73 expression is directly repressed by ZEB binding, establishing a link between the ZEB proteins and the p53 family. In addition, TAp73 isoforms were also found to be repressed to a lesser extent during myoblast differentiation and in mouse embryonic fibroblasts (MEFs) [134] (Figure 4).

In hepatocellular carcinoma (HCC), p53 upregulates the miR-200, miR-192, and other miRNAs [135]. The p53 protein inhibits EMT by downregulating the ZEB1/2 transcription factors. Furthermore, p53-regulated miR-200 and miR-192 family members were shown to be involved in a p53-adjusted EMT. Similarly, p53 knockdown could upregulate ZEB1 in epithelial cells, which in turn induced EMT and affected EMT-associated stem cell properties. On the other hand, p53 overexpression could reverse EMT and stem cell characteristics [136]. The work of Chang et al. reveals a role for p53 in regulating EMT-MET and stemness and implies a potential therapy of the suppression of cancer stem cells (CSCs), which is associated with EMT through the activation of the p53-miR-200 pathway. The above-mentioned findings elucidate a new function of p53 in which it ensures the epithelial properties. Although the p63/p73 genes are overexpressed as different isoforms, the p53 gene is usually mutated in a majority of carcinomas. Thus, in the absence of p53, p63 and p73 may be involved in the control of the ZEB/miR-200 loop. In fact, both proteins have been identified as positive regulators of miR-200 in ovarian carcinoma cells by directly modulating its promoter activity [137].

## 7. EMT as a Characteristic of Cancer Stem Cells (CSCs)

The term cancer stem cell was adopted in 2006 [138] to define the population of cancer cells with the ability to self-renew and differentiate just like the stem cells. The name, cancer stem cell, was invented to represent its properties of self-renewal and multipotency. CSCs can self-renew but it is debatable whether they can differentiate into multiple types of cells. Also, the term CSCs implies that these cells have originated from normal stem cells. The stem cells may be a source of origin of CSCs, but they are certainly not the only source [139]. For these reasons, many prefer to call them tumor initiating cells.

Many studies have recently linked the CSC phenotype to tumor cells undergoing EMT [140–148]. Morel et al. [141] recently showed that nontumorigenic mammary epithelial cells can give rise to a cell population that displays CD44+CD24− stem-like signatures through the activation of the RAS/MAPK pathway. This cell population displays an EMT phenotype that is characterized by the loss of E-cadherin and gain of vimentin expression [140, 141]. The linkage between EMT and stemness is further supported by the finding that Snail1 or Twist expression resulted in the loss of epithelial phenotype and the acquisition of mesenchymal phenotype in mammary epithelial cells and that the constitutive expression of either protein increased tumor initiating potential in transformed mammary epithelial cells [149, 150].

In prostate cancers, invasive cells exhibited CSC-like characteristics in an in vitro study of established and primary prostate cell lines. These cells were more tumorigenic than their counterparts and had a higher expression of the surface marker CD44, as well as of genes involved in the maintenance of a stem cell phenotype, including Nanog [151]. Although these authors did not investigate spontaneous metastasis by the invasive cells in vivo, Mulholland et al. [152] showed that EMT promotes the metastasis of cells with CSC characteristics. They engineered a mouse model of prostate cancer that better reflects the human situation, notably in terms of metastasis. The authors showed that RAS activation in PTEN-null cells resulted in EMT and that these EMT-induced cells had CSC characteristics and were responsible for micro- and macrometastases [152]. Similar results linking EMT and stemness were recently reported for putative CSCs from cervical cancer cell lines [153].

## 8. miRNA-Based Therapeutics and Clinical Trials

We have now entered the era that miRNAs are in trials to be used as a therapeutic tool against cancer. Depending on their pro- or antitumoral properties, different strategies based on blocking miRNA function or specific miRNA delivery to the tumor cells can be used. Several preclinical approaches have been developed in order to block miRNAs, including anti-miRNA oligonucleotides, miRNA sponges, miRNA masks (target protectors), and small molecule inhibitors [154].

Despite the challenges presented in delivering these molecules to the cells, there are currently two clinical trials for miRNA-based therapeutics [155]. Targeting miRNAs may be used directly to target tumor cells and also to enhance other therapies. For example, they could be used in reducing the drug resistance of tumors as has been shown by the chemoresistant properties of miR-100 in NSCLC [156] and the epigenetic silencing of miR-199b-5p in chemoresistant ovarian carcinoma [157].

The most advanced miRNA trial involves the use of anti-miR-122 (miravirsen) for hepatitis C therapy [158], which shows reduction in viral RNA with no evidence of resistance. Miravirsen is complementary to miR-122 but has also a modified locked-nucleic acid (LNA) structure which provides resistance to degradation and increased affinity for its target. Apart from targeting the mature miR-122, miravirsen was shown to target both the pri- and pre-miR-122 forms, thus leading to reduced processing and enhancement of its therapeutic effect [159].

The first miRNA-based therapy for cancer is MIRX34. It entered clinical testing in 2013 and is currently being studied in a multicenter, open-label Phase 1 clinical trial in patients with unresectable primary liver cancer or solid cancers with liver involvement. The trial also includes a separate cohort of patients with hematological malignancies. MRX34 was designed to deliver a mimic of the naturally occurring tumor suppressor, miR-34, which is lost or underexpressed in tumors of patients with a wide variety of cancers, including cervical cancer, ovarian cancer, glioblastoma, hepatocellular carcinoma (liver cancer), colon cancer, and non-small-cell lung cancer (NSCLC) and in cancer stem cells [160–162]. The miR-34 mimic is encapsulated using an innovative liposomal formulation called SMARTICLES.

The MRX34 Phase 1 clinical study in liver-based cancers is expected to be completed at the end of the first quarter of 2015, while the top-line results from the hematological malignancy cohort are expected in mid-2015. The primary objectives of the clinical trial are to establish the maximum tolerated dose and the recommended Phase 2 dose for future clinical trials. The secondary objectives are to assess the safety, tolerability, and pharmacokinetic profile of MRX34 as well as assess any biological activity and clinical outcomes. Nevertheless, miRNA-therapeutics is still in its infancy and the side effects of these therapies need to be carefully evaluated.

## 9. Conclusion

EMT plays a major role in cancer metastasis and is a complex, multifunctional, and tightly regulated developmental program. Understanding the different strategies employed by tumor cells to switch EMT on and off and the biological functions of the increasing number of the newly discovered miRNAs will lead to the development of new strategies in the diagnosis, prognosis, and treatment of human cancers. We have now entered a new exciting era, where clinical trials utilizing miRNA profiling for patient prognosis and clinical response are underway, and the first miRNA mimic has already entered the clinic for cancer therapy.

## Abbreviations

TGF-*β*: Transforming growth factor-beta
RTKs: Receptor tyrosine kinases
PDGF: Platelet-derived growth factor
FGFRs: Fibroblast growth factor receptors
PI3K: Phosphatidylinositol-3-kinase
NF-*κ*B: Nuclear factor kappa-light-chain-enhancer of activated B cells
JNK: c-Jun N-terminal kinase
ERK: Ras/Raf/Mitogen-activated protein kinase/ERK kinase (MEK)/extracellular-signal-regulated kinase
GEF: Guanine nucleotide exchange factor
EMT: Epithelial to mesenchymal transition
MET: Mesenchymal to epithelial transition
miRISC: miRNA-induced silencing complex.

## References

[1] Duband J. L., Monier F., Delannet M., Newgreen D. (1995). Epithelium-mesenchyme transition during neural crest development. *Acta Anatomica*.

[2] Bolender D. L., Markwald R. R. (1979). Epithelial-mesenchymal transformation in chick atrioventricular cushion morphogenesis. *Scanning Electron Microscopy*.

[3] Griffith C. M., Hay E. D. (1992). Epithelial-mesenchymal transformation during palatal fusion: carboxyfluorescein traces cells at light and electron microscopic levels. *Development*.

[4] Yilmaz M., Christofori G. (2009). EMT, the cytoskeleton, and cancer cell invasion. *Cancer and Metastasis Reviews*.

[5] Kalluri R., Weinberg R. A. (2009). The basics of epithelial-mesenchymal transition. *Journal of Clinical Investigation*.

[6] Soomets U., Hällbrink M., Langel U. (1999). Antisense properties of peptide nucleic acids. *Frontiers in Bioscience*.

[7] Thiery J. P., Sleeman J. P. (2006). Complex networks orchestrate epithelial-mesenchymal transitions. *Nature Reviews Molecular Cell Biology*.

[8] Their J. P. (2002). Epithelial-mesenchymal transitions in tumor progression. *Nature Reviews Cancer*.

[9] Hansen S. M., Berezin V., Bock E. (2008). Signaling mechanisms of neurite outgrowth induced by the cell adhesion molecules NCAM and N-Cadherin. *Cellular and Molecular Life Sciences*.

[10] Ouyang G., Wang Z., Fang X., Liu J., Yang C. J. (2010). Molecular signaling of the epithelial to mesenchymal transition in generating and maintaining cancer stem cells. *Cellular and Molecular Life Sciences*.

[11] Thiery J. P., Acloque H., Huang R. Y. J., Nieto M. A. (2009). Epithelial-mesenchymal transitions in development and disease. *Cell*.

[12] Spaderna S., Schmalhofer O., Wahlbuhl M. (2008). The transcriptional repressor ZEB1 promotes metastasis and loss of cell polarity in cancer. *Cancer Research*.

[13] Wood L. D., Parsons D. W., Jones S. (2007). The genomic landscapes of human breast and colorectal cancers. *Science*.

[14] Jones S., Zhang X., Parsons D. W. (2008). Core signaling pathways in human pancreatic cancers revealed by global genomic analyses. *Science*.

[15] Lamouille S., Xu J., Derynck R. (2014). Molecular mechanisms of epithelial-mesenchymal transition. *Nature Reviews Molecular Cell Biology*.

[16] Fuxe J., Vincent T., de Herreros A. G. (2010). Transcriptional crosstalk between TGF*β* and stem cell pathways in tumor cell invasion: role of EMT promoting Smad complexes. *Cell Cycle*.

[17] Zavadil J., Böttinger E. P. (2005). TGF-*β* and epithelial-to-mesenchymal transitions. *Oncogene*.

[18] Gotzmann J., Mikula M., Eger A. (2004). Molecular aspects of epithelial cell plasticity: implications for local tumor invasion and metastasis. *Mutation Research—Reviews in Mutation Research*.

[19] Wang Y., Zhou B. P. (2011). Epithelial-mesenchymal transition in breast cancer progression and metastasis. *Chinese Journal of Cancer*.

[20] Garg M. (2013). Epithelial-mesenchymal transition-activating transcription factors-multifunctional regulators in cancer. *World Journal of Stem Cells*.

[21] MacDonald B. T., Tamai K., He X. (2009). Wnt/*β*-catenin signaling: components, mechanisms, and diseases. *Developmental Cell*.

[22] Rao T. P., Kühl M. (2010). An updated overview on wnt signaling pathways: a prelude for more. *Circulation Research*.

[23] Sarkar F. H., Li Y., Wang Z., Kong D. (2010). The role of nutraceuticals in the regulation of Wnt and Hedgehog signaling in cancer. *Cancer and Metastasis Reviews*.

[24] Miele L. (2006). Notch signaling. *Clinical Cancer Research*.

[25] Wang Z., Li Y., Kong D., Sarkar F. H. (2010). The role of notch signaling pathway in Epithelial-Mesenchymal Transition (EMT) during development and tumor aggressiveness. *Current Drug Targets*.

[26] Bray S. J. (2006). Notch signalling: a simple pathway becomes complex. *Nature Reviews Molecular Cell Biology*.

[27] Leong K. G., Niessen K., Kulic I. (2007). Jagged1-mediated Notch activation induces epithelial-to-mesenchymal transition through Slug-induced repression of E-cadherin. *Journal of Experimental Medicine*.

[28] Collu G. M., Brennan K. (2007). Cooperation between Wnt and Notch signalling in human breast cancer. *Breast Cancer Research*.

[29] Wheelock M. J., Johnson K. R. (2003). Cadherin-mediated cellular signaling. *Current Opinion in Cell Biology*.

[30] Zeisberg M., Neilson E. G. (2009). Biomarkers for epithelial-mesenchymal transitions. *The Journal of Clinical Investigation*.

[31] Wang Q., Wang Y., Zhang Y., Xiao W. (2013). The role of uPAR in epithelial-mesenchymal transition in small airway epithelium of patients with chronic obstructive pulmonary disease. *Respiratory Research*.

[32] Chandrasekar N., Mohanam S., Gujrati M., Olivero W. C., Dinh D. H., Rao J. S. (2003). Downregulation of uPA inhibits migration and PI3k/Akt signaling in glioblastoma cells. *Oncogene*.

[33] Jo M., Lester R. D., Montel V., Eastman B., Takimoto S., Gonias S. L. (2009). Reversibility of epithelial-mesenchymal transition (EMT) induced in breast cancer cells by activation of urokinase receptor-dependent cell signaling. *The Journal of Biological Chemistry*.

[34] Lester R. D., Jo M., Montel V., Takimoto S., Gonias S. L. (2007). uPAR induces epithelial-mesenchymal transition in hypoxic breast cancer cells. *Journal of Cell Biology*.

[35] Larue L., Bellacosa A. (2005). Epithelial-mesenchymal transition in development and cancer: role of phosphatidylinositol 3′ kinase/AKT pathways. *Oncogene*.

[36] Kozomara A., Griffiths-Jones S. (2011). MiRBase: integrating microRNA annotation and deep-sequencing data. *Nucleic Acids Research*.

[37] Krol J., Loedige I., Filipowicz W. (2010). The widespread regulation of microRNA biogenesis, function and decay. *Nature Reviews Genetics*.

[38] Dalmay T. (2013). Mechanism of miRNA-mediated repression of mRNA translation. *Essays in Biochemistry*.

[39] Zaravinos A., Lambrou G. I., Mourmouras N. (2014). New miRNA profiles accurately distinguish renal cell carcinomas and upper tract urothelial carcinomas from the normal kidney. *PLoS ONE*.

[40] Radojicic J., Zaravinos A., Vrekoussis T., Kafousi M., Spandidos D. A., Stathopoulos E. N. (2011). MicroRNA expression analysis in triple-negative (ER, PR and Her2/neu) breast cancer. *Cell Cycle*.

[41] Chaveles I., Zaravinos A., Habeos I. G. (2012). MicroRNA profiling in murine liver after partial hepatectomy. *International Journal of Molecular Medicine*.

[42] Chartoumpekis D. V., Zaravinos A., Ziros P. G. (2012). Differential expression of microRNAs in adipose tissue after long-term high-fat diet-induced obesity in mice. *PLoS ONE*.

[43] Zaravinos A., Radojicic J., Lambrou G. I. (2012). Expression of miRNAs involved in angiogenesis, tumor cell proliferation, tumor suppressor inhibition, epithelial-mesenchymal transition and activation of metastasis in bladder cancer. *Journal of Urology*.

[44] Rana T. M. (2007). Illuminating the silence: understanding the structure and function of small RNAs. *Nature Reviews Molecular Cell Biology*.

[45] Zhang B., Wang Q., Pan X. (2007). MicroRNAs and their regulatory roles in animals and plants. *Journal of Cellular Physiology*.

[46] Filipowicz W., Jaskiewicz L., Kolb F. A., Pillai R. S. (2005). Post-transcriptional gene silencing by siRNAs and miRNAs. *Current Opinion in Structural Biology*.

[47] Hausser J., Syed A. P., Bilen B., Zavolan M. (2013). Analysis of CDS-located miRNA target sites suggests that they can effectively inhibit translation. *Genome Research*.

[48] Zhang B., Pan X., Cobb G. P., Anderson T. A. (2007). microRNAs as oncogenes and tumor suppressors. *Developmental Biology*.

[49] Ohlsson Teague E. M. C., Print C. G., Hull M. L. (2009). The role of microRNAs in endometriosis and associated reproductive conditions. *Human Reproduction Update*.

[50] Koutsaki M., Spandidos D. A., Zaravinos A. (2014). Epithelial-mesenchymal transition-associated miRNAs in ovarian carcinoma, with highlight on the miR-200 family: prognostic value and prospective role in ovarian cancer therapeutics. *Cancer Letters*.

[51] Feng X., Wang Z., Fillmore R., Xi Y. (2014). MiR-200, a new star miRNA in human cancer. *Cancer Letters*.

[52] Díaz-López A., Moreno-Bueno G., Cano A. (2014). Role of microRNA in epithelial to mesenchymal transition and metastasis and clinical perspectives. *Cancer Management and Research*.

[53] Adam L., Zhong M., Choi W. (2009). miR-200 expression regulates epithelial-to-mesenchymal transition in bladder cancer cells and reverses resistance to epidermal growth factor receptor therapy. *Clinical Cancer Research*.

[54] Tryndyak V. P., Beland F. A., Pogribny I. P. (2010). E-cadherin transcriptional down-regulation by epigenetic and microRNA-200 family alterations is related to mesenchymal and drug-resistant phenotypes in human breast cancer cells. *International Journal of Cancer*.

[55] Wiklund E. D., Bramsen J. B., Hulf T. (2011). Coordinated epigenetic repression of the miR-200 family and miR-205 in invasive bladder cancer. *International Journal of Cancer*.

[56] Dykxhoorn D. M., Wu Y., Xie H. (2009). miR-200 enhances mouse breast cancer cell colonization to form distant metastases. *PLoS ONE*.

[57] Elson-Schwab I., Lorentzen A., Marshall C. J. (2010). MicroRNA-200 family members differentially regulate morphological plasticity and mode of melanoma cell invasion. *PLoS ONE*.

[58] Hu X., Macdonald D. M., Huettner P. C. (2009). A miR-200 microRNA cluster as prognostic marker in advanced ovarian cancer. *Gynecologic Oncology*.

[59] Ali S., Ahmad A., Banerjee S. (2010). Gemcitabine sensitivity can be induced in pancreatic cancer cells through modulation of miR-200 and miR-21 expression by curcumin or its analogue CDF. *Cancer Research*.

[60] Li Y., Vandenboom T. G., Kong D. (2009). Up-regulation of miR-200 and let-7 by natural agents leads to the reversal of epithelial-to-mesenchymal transition in gemcitabine-resistant pancreatic cancer cells. *Cancer Research*.

[61] Kong D., Li Y., Wang Z. (2009). miR-200 regulates PDGF-D-mediated epithelial-mesenchymal transition, adhesion, and invasion of prostate cancer cells. *Stem Cells*.

[62] Shinozaki A., Sakatani T., Ushiku T. (2010). Downregulation of microRNA-200 in EBV-associated gastric carcinoma. *Cancer Research*.

[63] Gibbons D. L., Lin W., Creighton C. J. (2009). Contextual extracellular cues promote tumor cell EMT and metastasis by regulating miR-200 family expression. *Genes and Development*.

[64] Gregory P. A., Bert A. G., Paterson E. L. (2008). The miR-200 family and miR-205 regulate epithelial to mesenchymal transition by targeting ZEB1 and SIP1. *Nature Cell Biology*.

[65] Park S.-M., Gaur A. B., Lengyel E., Peter M. E. (2008). The miR-200 family determines the epithelial phenotype of cancer cells by targeting the E-cadherin repressors ZEB1 and ZEB2. *Genes & Development*.

[66] Pacurari M., Addison J. B., Bondalapati N. (2013). The microRNA-200 family targets multiple non-small cell lung cancer prognostic markers in H1299 cells and BEAS-2B cells. *International Journal of Oncology*.

[67] Schliekelman M. J., Gibbons D. L., Faca V. M. (2011). Targets of the tumor suppressor miR-200 in regulation of the epithelial-mesenchymal transition in cancer. *Cancer Research*.

[68] Gregory P. A., Bracken C. P., Smith E. (2011). An autocrine TGF-*β*/ZEB/miR-200 signaling network regulates establishment and maintenance of epithelial-mesenchymal transition. *Molecular Biology of the Cell*.

[69] Vandewalle C., Van Roy F., Berx G. (2009). The role of the ZEB family of transcription factors in development and disease. *Cellular and Molecular Life Sciences*.

[70] Hill L., Browne G., Tulchinsky E. (2012). ZEB/miR-200 feedback loop: at the crossroads of signal transduction in cancer. *International Journal of Cancer*.

[71] Hurteau G. J., Carlson J. A., Spivack S. D., Brock G. J. (2007). Overexpression of the MicroRNA hsa-miR-200c leads to reduced expression of transcription factor 8 and increased expression of E-cadherin. *Cancer Research*.

[72] Korpal M., Lee E. S., Hu G., Kang Y. (2008). The miR-200 family inhibits epithelial-mesenchymal transition and cancer cell migration by direct targeting of E-cadherin transcriptional repressors ZEB1 and ZEB2. *The Journal of Biological Chemistry*.

[73] Burk U., Schubert J., Wellner U. (2008). A reciprocal repression between ZEB1 and members of the miR-200 family promotes EMT and invasion in cancer cells. *EMBO Reports*.

[74] Bracken C. P., Gregory P. A., Kolesnikoff N. (2008). A double-negative feedback loop between ZEB1-SIP1 and the microRNA-200 family regulates epithelial-mesenchymal transition. *Cancer Research*.

[75] Brabletz S., Brabletz T. (2010). The ZEB/miR-200 feedback loop—a motor of cellular plasticity in development and cancer?. *EMBO Reports*.

[76] Kurashige J., Kamohara H., Watanabe M. (2012). MicroRNA-200b regulates cell proliferation, invasion, and migration by directly targeting ZEB2 in gastric carcinoma. *Annals of Surgical Oncology*.

[77] Cong N., Du P., Zhang A. (2013). Downregulated microRNA-200a promotes EMT and tumor growth through the Wnt/*β*-catenin pathway by targeting the E-cadherin repressors ZEB1/ZEB2 in gastric adenocarcinoma. *Oncology Reports*.

[78] Grünert S., Jechlinger M., Beug H. (2003). Diverse cellular and molecular mechanisms contribute to epithelial plasticity and metastasis. *Nature Reviews Molecular Cell Biology*.

[79] Jechlinger M., Sommer A., Moriggl R. (2006). Autocrine PDGFR signaling promotes mammary cancer metastasis. *Journal of Clinical Investigation*.

[80] Gotzmann J., Fischer A. N., Zojer M. (2006). A crucial function of PDGF in TGF-*β*-mediated cancer progression of hepatocytes. *Oncogene*.

[81] Lehmann K., Janda E., Pierreux C. E. (2000). Raf induces TGF*β* production while blocking its apoptotic but not invasive responses: a mechanism leading to increased malignancy in epithelial cells. *Genes and Development*.

[82] Eger A., Stockinger A., Schaffhauser B., Beug H., Foisner R. (2000). Epithelial mesenchymal transition by c-Fos estrogen receptor activation involves nuclear translocation of *β*-catenin and upregulation of *β*-catenin/lymphoid enhancer binding factor-1 transcriptional activity. *Journal of Cell Biology*.

[83] Lu J., Guo H., Treekitkarnmongkol W. (2009). 14-3-3*ζ* cooperates with ErbB2 to promote ductal carcinoma in situ progression to invasive breast cancer by inducing epithelial-mesenchymal transition. *Cancer Cell*.

[84] Braun J., Hoang-Vu C., Dralle H., Hüttelmaier S. (2010). Downregulation of microRNAs directs the EMT and invasive potential of anaplastic thyroid carcinomas. *Oncogene*.

[85] Carstens J. L., Lovisa S., Kalluri R. (2014). Microenvironment-dependent cues trigger miRNA-regulated feedback loop to facilitate the EMT/MET switch. *Journal of Clinical Investigation*.

[86] Berx G., Raspé E., Christofori G., Thiery J. P., Sleeman J. P. (2007). Pre-EMTing metastasis? Recapitulation of morphogenetic processes in cancer. *Clinical & Experimental Metastasis*.

[87] Sempere L. F., Christensen M., Silahtaroglu A. (2007). Altered microRNA expression confined to specific epithelial cell subpopulations in breast cancer. *Cancer Research*.

[88] Gregory P. A., Bracken C. P., Bert A. G., Goodall G. J. (2008). MicroRNAs as regulators of epithelial-mesenchymal transition. *Cell Cycle*.

[89] Kato M., Zhang J., Wang M. (2007). MicroRNA-192 in diabetic kidney glomeruli and its function in TGF-*β*-induced collagen expression via inhibition of E-box repressors. *Proceedings of the National Academy of Sciences of the United States of America*.

[90] Ru P., Steele R., Newhall P., Phillips N. J., Toth K., Ray R. B. (2012). miRNA-29b suppresses prostate cancer metastasis by regulating epithelial-mesenchymal transition signaling. *Molecular Cancer Therapeutics*.

[91] Zhang J., Zhang H., Liu J. (2012). MiR-30 inhibits TGF-*β*1-induced epithelial-to-mesenchymal transition in hepatocyte by targeting Snail1. *Biochemical and Biophysical Research Communications*.

[92] Kumarswamy R., Mudduluru G., Ceppi P. (2012). MicroRNA-30a inhibits epithelial-to-mesenchymal transition by targeting Snai1 and is downregulated in non-small cell lung cancer. *International Journal of Cancer*.

[93] Zhang J. P., Zeng C., Xu L., Gong J., Fang J. H., Zhuang S. M. (2014). MicroRNA-148a suppresses the epithelial-mesenchymal transition and metastasis of hepatoma cells by targeting Met/Snail signaling. *Oncogene*.

[94] Siemens H., Jackstadt R., Hünten S. (2011). miR-34 and SNAIL form a double-negative feedback loop to regulate epithelial-mesenchymal transitions. *Cell Cycle*.

[95] Moes M., Le Béchec A., Crespo I. (2012). A novel network integrating a mirna-203/snai1 feedback loop which regulates epithelial to mesenchymal transition. *PLoS ONE*.

[96] Ma L., Teruya-Feldstein J., Weinberg R. A. (2007). Tumour invasion and metastasis initiated by microRNA-10b in breast cancer. *Nature*.

[97] Lenferink A. E. G., Cantin C., Nantel A. (2010). Transcriptome profiling of a TGF-*β*-induced epithelial-to-mesenchymal transition reveals extracellular clusterin as a target for therapeutic antibodies. *Oncogene*.

[98] Turcatel G., Rubin N., El-Hashash A., Warburton D. (2012). Mir-99a and mir-99b modulate TGF-*β* induced epithelial to mesenchymal plasticity in normal murine mammary gland cells. *PLoS ONE*.

[99] Kong W., Yang H., He L. (2008). MicroRNA-155 is regulated by the transforming growth factor *β*/Smad pathway and contributes to epithelial cell plasticity by targeting RhoA. *Molecular and Cellular Biology*.

[100] Gebeshuber C. A., Zatloukal K., Martinez J. (2009). miR-29a suppresses tristetraprolin, which is a regulator of epithelial polarity and metastasis. *EMBO Reports*.

[101] Cottonham C. L., Kaneko S., Xu L. (2010). miR-21 and miR-31 converge on TIAM1 to regulate migration and invasion of colon carcinoma cells. *Journal of Biological Chemistry*.

[102] Eades G., Yao Y., Yang M., Zhang Y., Chumsri S., Zhou Q. (2011). miR-200a regulates SIRT1 expression and epithelial to mesenchymal transition (EMT)-like transformation in mammary epithelial cells. *Journal of Biological Chemistry*.

[103] Eis P. S., Tam W., Sun L. (2005). Accumulation of miR-155 and BIC RNA in human B cell lymphomas. *Proceedings of the National Academy of Sciences of the United States of America*.

[104] Slaby O., Svoboda M., Fabian P. (2008). Altered expression of miR-21, miR-31, miR-143 and miR-145 is related to clinicopathologic features of colorectal cancer. *Oncology*.

[105] Valastyan S., Chang A., Benaich N., Reinhardt F., Weinberg R. A. (2011). Activation of miR-31 function in already-established metastases elicits metastatic regression. *Genes and Development*.

[106] Ma L., Young J., Prabhala H. (2010). MiR-9, a MYC/MYCN-activated microRNA, regulates E-cadherin and cancer metastasis. *Nature Cell Biology*.

[107] Ceteci F., Ceteci S., Karreman C. (2007). Disruption of tumor cell adhesion promotes angiogenic switch and progression to micrometastasis in RAF-driven murine lung cancer. *Cancer Cell*.

[108] Meng Z., Fu X., Chen X. (2010). miR-194 is a marker of hepatic epithelial cells and suppresses metastasis of liver cancer cells in mice. *Hepatology*.

[109] Zhang L.-Y., Liu M., Li X., Tang H. (2013). MiR-490-3p modulates cell growth and epithelial to mesenchymal transition of hepatocellular carcinoma cells by targeting endoplasmic reticulum-golgi intermediate compartment protein 3 (ERGIC3). *Journal of Biological Chemistry*.

[110] Nishikawa M., Kira Y., Yabunaka Y., Inoue M. (2007). Identification and characterization of endoplasmic reticulum-associated protein, ERp43. *Gene*.

[111] Wang F. E., Zhang C., Maminishkis A. (2010). MicroRNA-204/211 alters epithelial physiology. *FASEB Journal*.

[112] Barrios A., Poole R. J., Durbin L., Brennan C., Holder N., Wilson S. W. (2003). Eph/Ephrin signaling regulates the mesenchymalto-epithelial transition of the paraxial mesoderm during somite morphogenesis. *Current Biology*.

[113] Fukai J., Yokote H., Yamanaka R., Arao T., Nishio K., Itakura T. (2008). EphA4 promotes cell proliferation and migration through a novel EphA4-FGFR1 signaling pathway in the human glioma U251 cell line. *Molecular Cancer Therapeutics*.

[114] Yan Y., Luo Y.-C., Wan H.-Y. (2013). MicroRNA-10a is involved in the metastatic process by regulating Eph tyrosine kinase receptor A4-mediated epithelial-mesenchymal transition and adhesion in hepatoma cells. *Hepatology*.

[115] Stinson S., Lackner M. R., Adai A. T. (2011). TRPS1 targeting by miR-221/222 promotes the epithelial-to-mesenchymal transition in breast cancer. *Science Signaling*.

[116] Gai Z., Zhou G., Itoh S. (2009). Trps1 functions downstream of Bmp7 in kidney development. *Journal of the American Society of Nephrology*.

[117] Shah M. Y., Calin G. A. (2011). MicroRNAs miR-221 and miR-222: a new level of regulation in aggressive breast cancer. *Genome Medicine*.

[118] Guttilla I. K., Phoenix K. N., Hong X., Tirnauer J. S., Claffey K. P., White B. A. (2012). Prolonged mammosphere culture of MCF-7 cells induces an EMT and repression of the estrogen receptor by microRNAs. *Breast Cancer Research and Treatment*.

[119] Koch U., Radtke F. (2007). Notch and cancer: a double-edged sword. *Cellular and Molecular Life Sciences*.

[120] Jiang L., Liu X., Kolokythas A. (2010). Downregulation of the Rho GTPase signaling pathway is involved in the microRNA-138-mediated inhibition of cell migration and invasion in tongue squamous cell carcinoma. *International Journal of Cancer*.

[121] Liu X., Wang C., Chen Z. (2011). MicroRNA-138 suppresses epithelial-mesenchymal transition in squamous cell carcinoma cell lines. *Biochemical Journal*.

[122] Konno Y., Dong P., Xiong Y. (2014). MicroRNA-101 targets EZH2, MCL-1 and FOS to suppress proliferation, invasion and stem cell-like phenotype of aggressive endometrial cancer cells. *Oncotarget*.

[123] Wang Z., Li Y., Kong D. (2009). Acquisition of epithelial-mesenchymal transition phenotype of gemcitabine-resistant pancreatic cancer cells is linked with activation of the notch signaling pathway. *Cancer Research*.

[124] Brabletz S., Bajdak K., Meidhof S. (2011). The ZEB1/miR-200 feedback loop controls Notch signalling in cancer cells. *The EMBO Journal*.

[125] Vallejo D. M., Caparros E., Dominguez M. (2011). Targeting Notch signalling by the conserved miR-8/200 microRNA family in development and cancer cells. *The EMBO Journal*.

[126] Spaderna S., Schmalhofer O., Hlubek F. (2006). A transient, EMT-linked loss of basement membranes indicates metastasis and poor survival in colorectal cancer. *Gastroenterology*.

[127] Sánchez-Tilló E., de Barrios O., Siles L., Cuatrecasas M., Castells A., Postigo A. (2011). *β*-catenin/TCF4 complex induces the epithelial-to-mesenchymal transition (EMT)-activator ZEB1 to regulate tumor invasiveness. *Proceedings of the National Academy of Sciences of the United States of America*.

[128] Shi Y., Massagué J. (2003). Mechanisms of TGF-*β* signaling from cell membrane to the nucleus. *Cell*.

[129] Hermeking H. (2007). p53 enters the microRNA world. *Cancer Cell*.

[130] Chari N. S., Pinaire N. L., Thorpe L., Medeiros L. J., Routbort M. J., McDonnell T. J. (2009). The p53 tumor suppressor network in cancer and the therapeutic modulation of cell death. *Apoptosis*.

[131] Lewis B. C., Klimstra D. S., Socci N. D., Xu S., Koutcher J. A., Varmus H. E. (2005). The absence of *p53* promotes metastasis in a novel somatic mouse model for hepatocellular carcinoma. *Molecular and Cellular Biology*.

[132] Chen Y.-W., Klimstra D. S., Mongeau M. E., Tatem J. L., Boyartchuk V., Lewis B. C. (2007). Loss of p53 and Ink4a/Arf cooperate in a cell autonomous fashion to induce metastasis of hepatocellular carcinoma cells. *Cancer Research*.

[133] Hansen J. E., Fischer L. K., Chan G. (2007). Antibody-mediated p53 protein therapy prevents liver metastasis *in vivo*. *Cancer Research*.

[134] Fontemaggi G., Gurtner A., Strano S. (2001). The transcriptional repressor ZEB regulates p73 expression at the crossroad between proliferation and differentiation. *Molecular and Cellular Biology*.

[135] Kim T., Veronese A., Pichiorri F. (2011). p53 regulates epithelial-mesenchymal transition through microRNAs targeting ZEB1 and ZEB2. *Journal of Experimental Medicine*.

[136] Chang C.-J., Chao C.-H., Xia W. (2011). p53 regulates epithelial- mesenchymal transition and stem cell properties through modulating miRNAs. *Nature Cell Biology*.

[137] Knouf E. C., Garg K., Arroyo J. D. (2012). An integrative genomic approach identifies p73 and p63 as activators of miR-200 microRNA family transcription. *Nucleic Acids Research*.

[138] Clarke M. F., Dick J. E., Dirks P. B. (2006). Cancer stem cells—perspectives on current status and future directions: AACR workshop on cancer stem cells. *Cancer Research*.

[139] Chhabra R., Saini N. (2014). MicroRNAs in cancer stem cells: current status and future directions. *Tumor Biology*.

[140] Mani S. A., Guo W., Liao M.-J. (2008). The epithelial-mesenchymal transition generates cells with properties of stem cells. *Cell*.

[141] Morel A.-P., Lièvre M., Thomas C., Hinkal G., Ansieau S., Puisieux A. (2008). Generation of breast cancer stem cells through epithelial-mesenchymal transition. *PLoS ONE*.

[142] Hennessy B. T., Gonzalez-Angulo A. M., Stemke-Hale K. (2009). Characterization of a naturally occurring breast cancer subset enriched in epithelial-to-mesenchymal transition and stem cell characteristics. *Cancer Research*.

[143] Aktas B., Tewes M., Fehm T., Hauch S., Kimmig R., Kasimir-Bauer S. (2009). Stem cell and epithelial-mesenchymal transition markers are frequently overexpressed in circulating tumor cells of metastatic breast cancer patients. *Breast Cancer Research*.

[144] Hollier B. G., Evans K., Mani S. A. (2009). The epithelial-to-mesenchymal transition and cancer stem cells: a coalition against cancer therapies. *Journal of Mammary Gland Biology and Neoplasia*.

[145] Santisteban M., Reiman J. M., Asiedu M. K. (2009). Immune-induced epithelial to mesenchymal transition *in vivo* generates breast cancer stem cells. *Cancer Research*.

[146] Blick T., Hugo H., Widodo E. (2010). Epithelial mesenchymal transition traits in human breast cancer cell lines parallel the CD44HI/CD24lO/-stem cell phenotype in human breast cancer. *Journal of Mammary Gland Biology and Neoplasia*.

[147] Kong D., Banerjee S., Ahmad A. (2010). Epithelial to mesenchymal transition is mechanistically linked with stem cell signatures in prostate cancer cells. *PLoS ONE*.

[148] Zavadil J. (2010). A spotlight on regulatory networks connecting EMT and cancer stem cells. *Cell Cycle*.

[149] Martin A., Cano A. (2010). Tumorigenesis: twist1 links EMT to self-renewal. *Nature Cell Biology*.

[150] Yang M.-H., Hsu D. S.-S., Wang H.-W. (2010). Bmi1 is essential in Twist1-induced epithelial-mesenchymal transition. *Nature Cell Biology*.

[151] Klarmann G. J., Hurt E. M., Mathews L. A. (2009). Invasive prostate cancer cells are tumor initiating cells that have a stem cell-like genomic signature. *Clinical and Experimental Metastasis*.

[152] Mulholland D. J., Kobayashi N., Ruscetti M. (2012). Pten loss and RAS/MAPK activation cooperate to promote EMT and metastasis initiated from prostate cancer stem/progenitor cells. *Cancer Research*.

[153] Lopez J., Poitevin A., Mendoza-Martinez V., Perez-Plasencia C., Garcia-Carranca A. (2012). Cancer-initiating cells derived from established cervical cell lines exhibit stem-cell markers and increased radioresistance. *BMC Cancer*.

[154] Garzon R., Marcucci G., Croce C. M. (2010). Targeting microRNAs in cancer: rationale, strategies and challenges. *Nature Reviews Drug Discovery*.

[155] Bouchie A. (2013). First microRNA mimic enters clinic. *Nature Biotechnology*.

[156] Xiao F., Bai Y., Chen Z. (2014). Downregulation of HOXA1 gene affects small cell lung cancer cell survival and chemoresistance under the regulation of miR-100. *European Journal of Cancer*.

[157] Liu M. X., Siu M. K. Y., Liu S. S., Yam J. W. P., Ngan H. Y. S., Chan D. W. (2014). Epigenetic silencing of microRNA-199b-5p is associated with acquired chemoresistance via activation of JAG1-Notch1 signaling in ovarian cancer. *Oncotarget*.

[158] Janssen H. L. A., Reesink H. W., Lawitz E. J. (2013). Treatment of HCV infection by targeting microRNA. *The New England Journal of Medicine*.

[159] Gebert L. F. R., Rebhan M. A. E., Crivelli S. E. M., Denzler R., Stoffel M., Hall J. (2014). Miravirsen (SPC3649) can inhibit the biogenesis of miR-122. *Nucleic Acids Research*.

[160] Wiggins J. F., Ruffino L., Kelnar K. (2010). Development of a lung cancer therapeutic based on the tumor suppressor microRNA-34. *Cancer Research*.

[161] Bader A. G. (2012). MiR-34—a microRNA replacement therapy is headed to the clinic. *Frontiers in Genetics*.

[162] Daige C. L., Wiggins J. F., Priddy L. (2014). Systemic delivery of a miR34a mimic as a potential therapeutic for liver cancer. *Molecular Cancer Therapeutics*.

